# Human embryonic stem cells: preclinical perspectives

**DOI:** 10.1186/1479-5876-6-7

**Published:** 2008-01-29

**Authors:** Kaushik Dilip Deb, Kanchan Sarda

**Affiliations:** 1 Embryonic Stem Cell Program, Manipal Institute of Regenerative Medicine, #10 Service Road, Domlur, Bangalore 560071, India

## Abstract

Human embryonic stem cells (hESCs) have been extensively discussed in public and scientific communities for their potential in treating diseases and injuries. However, not much has been achieved in turning them into safe therapeutic agents. The hurdles in transforming hESCs to therapies start right with the way these cells are derived and maintained in the laboratory, and goes up-to clinical complications related to need for patient specific cell lines, gender specific aspects, age of the cells, and several post transplantation uncertainties. The different types of cells derived through directed differentiation of hESC and used successfully in animal disease and injury models are described briefly. This review gives a brief outlook on the present and the future of hESC based therapies, and talks about the technological advances required for a safe transition from laboratory to clinic.

## 1. Introduction

Biomedical research using embryonic stem cells (ESC) is a very promising area for the investigation of the possibilities of developing newer cell replacement therapies (CRTs). Diseases and disorders which have no therapy or at best partially effective ones mainly attract the pursuit of ESC research. The first ESC line was established from mouse embryos in 1981 [1], following a method virtually identical to rabbit models used by Cole RJ et al., [2] about 30 years earlier. These ESCs have been used for introducing specific gene modifications in mice. Sir Martin Evans has recently been honored with the Nobel Prize for Physiology and Medicine (2007) for his contribution towards development of animal models of disease through ESC mediated gene targeting. Human embryonic stem cells were first derived by Thompson's group in 1998 [3] and are usually derived from the inner cell mass (ICM) of blastocyst stage embryos that are left over after in vitro fertilization (IVF) and after embryo donations [4]. The derivation of hESCs have opened up huge possibilities for regeneration and repair of organs of tissues and thus been envisioned as therapeutic agents.

"Self-renewal" i.e., the ability to undergo indefinite self-renewing and symmetric cell divisions to maintain the population, and "pluripotency" which indicates their ability to differentiate into any of the 200 different known cell types (of ectoderm, endoderm, mesoderm and trophectoderm lineages); are the two hallmarks of these cells [5]. Though these are the key properties of the hESCs, yet they are not a property of the ICM *in vivo *and must be a characteristic adopted by the cells during their initial outgrowth in the culture. The use of human embryonic stem cells (hESCs) in cell replacement therapies (CRTs) has been limited due to several technical and ethical issues. Since its inception there has been an extensive debate about the benefits and drawbacks of adult vs hESC use in therapies [6,7]. For hESCs the problems range from the way they are derived, characterized, established and maintained, to their in-vitro differentiation and transplantation. Changes in their epigenetic profiles, chromosomal aberrations during their establishment and maintenance, post transplantation challenges like risk of tumors, genetic instability, and immune-rejections are some of the other major concerns.

ESC-like cells have also been derived from mouse skin cells [7]. ESC lines have also been established from single cell biopsies of a developing embryo [8,9]. Such advances if successfully reproduced in human, could possibly demolish the ethical objections related to destroying a potential life that has haunted the field for many years. This review presents a generalized opinion and outlook on the alternative strategies required to develop effective and novel hESCs based therapies.

## 2. Barriers to bringing hESCs to clinic

Seven major concerns identified as significant roadblocks to the safe transition of hESC to clinic are discussed in the following subsections.

### 2.1 Derivation of hESCs: need for "Embryo-friendly" ways

Destruction of life in the form of an embryo has been a major ethical objection in embryonic stem cell derivation and research in several western countries. One way to get around this objection will be to generate human ESC lines without the use of additional human embryos. The different approaches suggested so far are discussed below:

#### a) Reprogramming of adult cell nucleus

The method uses existing hESCs to fuse with an adult somatic cell, generating a cell line that retains ESC specific properties and yet has the genotype of the somatic cell donor [10]. However, there is no technology available to selectively remove all the ESC chromosomes while retaining the somatic cell chromosomes. In addition this removal of chromosome needs to be timed to occur only after the hybrid cell has been reprogrammed to take the properties of the stem cells. Development of such technologies is potentially expensive and will presumably take years.

#### b) ESCs from embryo like entities

This approach involves the use of somatic cell nuclear transfer (SCNT) to produce developmentally compromised embryo-like structures, with the help of genetically premodified deficient nuclei which cannot support development [11]. The zygote produced by such nuclear transfer undergoes cleavage in-vitro and produce ICM cells, which would be used for deriving ESCs, but would not proceed further in development. A proof of principle to this was accomplished by generating mouse ESCs, using a donor nucleus which was silenced for *Cdx2 *gene [12]. This is ethically correct for those who believe that fetal life begins only after the embryo implants. However, one need not go for creating a mutation to achieve this target, as a blastocyst cannot develop into a complete human life in vitro, irrespective of the presence or absence of any kind of genetic alterations.

#### c) ESC lines from single blastomeres

It is known that if a cell or two is missing from a preimplantation embryo it can regenerate the missing part and form a whole embryo. A single cell can be isolated from the cleavage stage embryo, a technique well established for preimplantation genetic diagnosis (PGDs), and used to create a cell line from it; the rest of the embryo can be transferred back to the uterus to give rise to a fetus [8]. Robert Lanza's group has shown that ESC lines could be established from single cell biopsies of the mouse and human embryos [8,9]. However, this technique is very difficult to translate to human being. Also, the fate of the residual embryos if they are transferred is largely unknown, as there is a lack of long term studies supporting the health of babies born following PGD.

#### d) ESC lines from induced somatic cell dedifferentiation

In this method the adult somatic cells are genetically modified and reprogrammed to undergo a process of dedifferentiation, by inducing the expression of pluripotency related genes. Recently, induced pluripotent stem cell lines have been derived by allowing trans-acting factors present in the mammalian oocytes to reprogram somatic cell nuclei to an undifferentiated state [13]. They have demonstrated that four factors OCT-4, SOX-2, Nanog and LIN28 are sufficient to reprogram human somatic stem cells to pluripotent stem cells. Whereas, Takahashi and Yamanaka (2006) induced somatic cells into pluripotent stem cells by introducing four factors OCT-4, SOX-2, c-Myc and KLF-4. These cells designated as induced pluripotent stem cells (iPS) exhibit morphology of embryonic stem cells and express ES cell markers [14]. Although, Takahashi and Yamanaka (2006) and Yu et al., (2007) carried out astonishing experiments by reprogramming somatic cells into pluripotent stem cells, several technical limitations such as use of retrovirus or lentiviruses for transfecting OCT-4, Nanog, SOX-2, C-MYC, LIN28 or KLF4 restrict the use of such cell lines for clinical applications [15] (Hanna et al., 2007).

#### e) Embryonic like stem cells from alternative sources

Adult stem cells similar to blastomeres of the preimplantation stage embryos have been identified and isolated by Henry Young and coworkers [16]. These cells called the blastomere-like stem cells (BLSCs) are found to be totipotent due to their potential to give rise to all tissue types including the gametes. These BLSCs can be induced to differentiate in a unidirectional manner to form pluripotent embryonic-like stem cells (ELSCs). It is also claimed that these cells do not express the MHC class-I or HLA DR-II cell surface markers. More recently Meng et al., (2007) have discovered a population of stem cells in the menstrual blood [17]. These cells named as the "Emdometrial Regenerative Cells" are shown to be capable of differentiating into 9 tissue lineages namely: cadiomyocytic, respiratory epithelial, neurocytic, myocytic, endothelial, pancreatic, hepatic, adipocytic, and osteogenic.

### 2.2 Need for xeno-free culture systems

Conventionally, human embryonic stem cell lines are grown 1) in a medium containing animal serum as a source of nutrients and growth factors and 2) on mouse-derived fibroblast as feeder layers, which play a role in the proliferation and inhibition of their differentiation. However, the use of any cell based therapeutic agent in human must be free of animal contaminations which may contain certain pathogens or xenogens that can trigger immune reactions after transfer to a host. Some laboratories have successfully cultured hESC in a serum-free defined medium on human cell-derived feeders or even in feeder free conditions [18 19]. Another significant achievement in this context was the derivation and establishment of a hESC line under animal product free condition [20].

Expression of a nonhuman sialic acid Neu5Gc and presence of murine viruses are two concerns in existing hESCs grown in presence of animal products or feeders [21]. Replacement of animal serum with human serum has been reported to reduce the expression of Neu5Gc in the hESCs, also Amit et al (2005) have reported the absence of murine leukemia virus in a number of hESC lines maintained on mouse feeders [22]. These studies are largely indicative of the fact that the problem of animal product contamination in the existing culture would be possibly solved in the near future.

### 2.3 Risk of tumors

Following transplantation hES cell based therapies involves the risk of tumor formation arising from undifferentiated population of the transplanted cells. Studies with both ESCs and ES derived differentiated cells have shown that they can form teratocarcinomas in adult mice if injected subcutaneously, intramuscularly or into the testis [5,8,23,24]. It has also not been possible to produce a pure population of more that 80% of differentiated cells from mouse or human ESCs using any of the directed differentiation protocols. In a cell culture for therapeutic use, the presence of even one undifferentiated cell may potentially lead to teratomas, a cancerous tumor which is derived from germ cells and can from all the three germ layers.

One way of obtaining a pure culture of differentiated cells would be to confirm a negative expression for Oct4 or Nanog in them. Also new strategies can be developed which in some way would tag the ESC implants that could accidentally forms tumors, with death or suicide signals [25]. Alternatively, ESCs can be genetically engineered so that a negative selection can be carried out based on a compound that is toxic to undifferentiated ESCs under certain culture conditions [26]. Studies with mouse ESC show that if a more differentiated cell population is used for grafting, the cells are less likely to generate a tumor [27]. A differentiated population of cells can be segregated from a mixed cell population using either a technique like Fluorescence Activated Cell Sorting (FACS) or other selective approaches [28,29]. Bieberich et al., (2004) showed that treating the mixed population with the ceramide analogue N-oleoyl serinol (S18) can selectively induce apoptosis of ESCs [30]. While adequate technologies for screening and generating a pure population of differentiated stem cells are available, it is yet not known whether they are capable of eliminating each and every potential tumor forming ES cell.

### 2.4 Genetic instability

Questions on the suitability of ESCs for transplantation purpose is raised because of the observed genetic instability of cloned cells and extreme inefficiency of the process [31]. Cloned animals like Dolly give the outward appearance of full health, but the probability of their having numerous genetic defects is very high. Hochedlinger and Rudolf Jaenisch (2002) showed that in mice, cloned using ESC in place of the somatic cells (which produces better results) the reprogramming of the inserted genetic material by the embryonic cells proceeded in a very unregulated way [32]. They reported that many of the genes that are necessary for the early phase of embryonic development were not activated.

### 2.5 Transplant rejection

The immune system tends to reject the transplanted ESCs as 'foreign'. This rejection can be inhibited by the use of immunosuppressive drugs which can have serious side effects. Alternate approaches using homolologous recombination techniques can allow the host immune system to recognize and mark the ESCs as 'self'. This is possible by replacing the MHC genes in ESCs with the host MHC genes [33]. Elimination of MHC class I and II gene loci is also proposed, though this would be technically challenging and would be clinically problematic because cells lacking MHC class I surface expression are targeted by NK cells [34,35].

One possible alternative is to establish a bank of MHC-compatible hESC lines. Taylor et al., (2005) reported that a reasonable HLA match for around 85% of the UK population could be achieved with around 150 lines [36]. However, this number might be conservative for countries like India and the USA, which have a varied and multi-racial population [37]. Even with such banks in most cases immunosuppressive drugs would still be needed for most donor patient combinations. Some also believe that the transplantation of hESC derivatives into immune-privileged sites such as the brain, say to treat Parkinson's disease, may possibly ameliorate the adverse effects of a MHC-mismatch, although the protection would only be short term.

A possible way to overcome immune rejection is to over express genes that can suppress the immune system such as fas-ligand into ES cells [38]. It is suggested that removal of certain cell surface molecules like B7 antigens or CD40 ligands that are immunologically reactive from ES cells prior to transplantation could suppress immune rejections. For this, somatic cell nucleus from the recipient/patient can be subjected to such genetic modifications and can be inserted into an enucleated oocyte. Though this technology would help in developing patient specific ESCs lines, it would also carry along with it several ethical and technical limitations [32]. As in most incurables diseases, if the patient is suffering from a degenerative condition which destroys their own cells, the transplanted ESCs will suffer the same fate. Therefore in such cases transplanting a population of cells that includes a proliferating progenitor is needed to provide a continuous source of differentiating cells. Again as discussed earlier these transplants might run the risk of tumor formation.

### 2.6 Epigenetic reprogramming and culture adaptation

HESC lines can differ from each other in their genomic expression profiles through epigenetic regulations. Two major causes for epigenetic changes in hESCs have been identified; 1) the epigenetic changes in preimplantation embryos used for derivation of the hES cell lines, and 2) epigenetic changes during their maintenance in the culture over time.

#### Epigenetic changes in embryos

In the preimplantation embryos epigenetic modifications such as DNA methylation and histone modifications are widely involved in the regulation of imprinted and non-imprinted genes. These events are often vulnerable to the external environment or culture conditions. Ericson and Kallen, (2001) have reported the occurrences of congenital malformations after the use of assisted reproductive technologies (ART) in human [39]. ART has also been linked to imprinting disorders, such as Beckwith-Wiedemann Syndrome and Angelman Syndrome (AS) [40,41]. It has been shown that these disorders result from hypomethylation of the maternal genome [42,43]. A set of a few such methylated and imprinted genes like TSSC5, H19, PEG1, SNRPN, Xist, Oct4, Notch1, DLK1 etc., are now being studied for their methylation status as a part of the characterization process for new hESC lines [44]. More elaborate studies on various existing hESC lines are required to identify the epigenetic markers for pluripotency. Most existing hESC lines have been established under different culture conditions, this might cause variations in epigenetic profiles over and above that inherited from in-vitro production of the embryo [45].

#### Epigenetic changes over time

Human ESCs can be used as suitable models for genetic defects, diseases, drug screening and cell replacement therapies only if they are genetically stable over long periods in culture. However, a recent report by Allergrucci et al., (2007), shows that hESCs can undergo epigenetic changes over time in culture [46]. They found maximum changes in the epigenetic profile of these cells at the early stages post derivation and after growing them in serum free culture systems [47]. In another report a DNA methylation profile for hESC was determined, and a set of 25 sites from 23 genes were also identified, which could distinguish normal hESCs from differentiated cells [48]. Lagarkowa et al., (2006) demonstrated that the methylation status of pluripotency genes like DPPA3 and DPPA5 varied between hESC lines and also during their differentiation into embryoid bodies [49]. All these studies indicate the need for optimization of procedures that would minimize culture-induced genomic instability. This also implies that periodic monitoring of these lines will be required to evaluate their suitability for in-vivo applications. It is possible that hESCs can get rapidly reprogrammed into unpredictable genetic changes and that some existing and late-passage hESC lines may not be suitable for therapeutic uses.

### 2.7 Chromosomal abnormalities during prolonged culture

Selection and adaptation of hESCs is a poorly understood phenomenon. An extreme variability in culture conditions exist not only between labs, but even within laboratories. The establishment and maintenance of these cells must involve some form of 'culture adaptation' process, most like epigenetic in nature as discussed above. However, several reports also indicate that these cells acquire chromosomal abnormalities or karyotype aberrations during prolonged culture in parallel with epigenetic changes [50]. Such adaptations may result in enhanced cloning efficiencies after plating single cells [51], a reduced tendency for apoptosis [52], and is expected to have a reduced capacity for differentiation which is difficult to assess quantitatively. A recent report by Baker et al., (2007) demonstrates clear evidence for the accumulation of specific chromosomal aberrations within several well-established hESC lines over time [53]. Results from previously published data were pooled and compared with their own data to access the temporal affects of long term *in vitro *maintenance on the genetic stability of hESC lines. The study indicates that hESCs indeed become chromosomally abnormal and 'culture adapted' in a reproducible, non-random nature over time. They also found a bias for gains in chromosomes 12, 17, and X, which are similar to chromosomal change seen in breast cancer and testicular germ cell tumor (TGCT) seminomas and non-seminomas. This indicates that these chromosomal regions may harbor replication dependent genes critical for cell proliferation. Interestingly, antiapoptotic genes like BIRC5; pluripotency genes like NANOG, DPPA3, and GDF3; cell-cycle regulator like CCND2; and other genes involved in cancer tumorigenesis like TGCT1, KRAS and SOX5, lie in chromosome 17, chromosome 12, and the X chromosome [53]. With all these evidences it is becoming increasingly clear that standard immuno-markers commonly employed to identify hESCs are not adequate to demonstrate that the hESCs are normal.

## 3. Embryonic stem cell based therapies: advances

What may have appeared to be impossible with ESC research several years ago is gradually turning into reality. Scientists are trying to coax the ES cells to differentiated population of cells which could be used for therapies. For a list of in vitro differentiated cell types derived from hESCs by various groups please refer to Deb et al., (2007) [54]. A list of preclinical animal models where human ESCs have demonstrated efficiency is listed in Table 1. Besides this scientists are trying to use ESCs for various other applications. For example, the use of ESCs as vehicles for tropic support for dying neurons is possibly a more feasible goal and many workers are focusing on this kind of studies [41]. Efforts are being made to use this technology, to modify the ESCs for use in delivery of genes and other factors to dying motor neurons.

**Table 1:** A list of animal injury and disease models where hESCs have been shown to be effective

**CELL TYPE DEVELOPED**	**ANIMAL MODEL**	**REFERENCE**
Oligodenrocyte progenitor	Spinal cord injury induced mouse	Keirstead et al., 2005 [69]; Nakamura et al., 2005 [70]
Cardiomyocytes	Rat, Swine, Mice	Laflamme et al., 2007 [71]; Leor et al., 1996 [72]; Kehat et al., 2004 [73]; Caspi et al., 2007 [74]
Hepatocyte	CCl4-injured SCID mouse model	Seo et al., 2005 [75]
Chondrocyte	Canine Spinal Fusion model	Muschler et al., 2003 [76]
Endothelial cells	Surgical induction of hind limb ischemia in athymic mouse	Cho et al., 2006 [77]
Neural precursors	Quinolinic acid (QA)-induced Huntington's disease (HD) model in rats	Song et al., 2007 [78]
Pancreatic cells	Streptozotocin-treated diabetic mice	Shim et al., 2007 [79]
Skeletal myoblasts	SCID/Beige mice	Barberi et al., 2007 [80]
Neuroepithelial precursors and Dopaminergic neurons	Parkinsons disease rodent model	Sonntag et al., 2007 [81] Ben-Hur et al., 2004 [82]
hESCs	Open neural tube defect (ONTD) model in chick embryos	Lee et al., 2006 [83]
T lymphoid lineage	Engraftment into human thymic tissues in immunodeficient mice	Galic et al., 2006 [84]

Generation of patient specific human nuclear transfer ESC (hNT-ESCs) lines is a strategy that may circumvent the problem of immuno-rejection which is the greatest challenge in CRTs [55]. The implications of transferring mitochondrial hetroplasmic cells, which might contain aberrant epigenetic gene expression profiles, are also of concern. Allogenic mitochondria present in the NT-ESC derived cells could be recognized by the host immune system, leading to disrupted mitochondrial membrane potential that induces apoptosis [52,56]. The mitochondrial genome is also known to encode a number of transplantation antigens that could trigger a immune response for the host tissue following engraftment [57], for example the maternally transmitted mtDNA-encoded minor histocompatibility antigen (mi-Has) [58,59]. This has been demonstrated in failed cardiomyocyte grafts with mitocondria induced apoptosis in a rat model [60].

Pathenogenetically activated embryos has been proposed for the creation of female haploid ESC lines. These cells could serve as an autologous source of cells for producing differentiated cell types to treat women suffering from diseases like Type 1 diabetes or spinal cord injuries [61]. This possibility has been tested in a primate model [62]. However, it is not know if a differentiated haploid cell type following a transplant would remain normal in-vivo. Recently Revazova et al., (2007) has reported the development of six patient specific stem cell lines from parthenogenetic blastocysts [63]. They have also used a protocol which minimizes the use of animal derived components to make the cell lines more suitable for clinical applications.

Despite the drawbacks and debates, an internet search shows some commercial sources offering embryonic stem cell therapy, like the Embryonic Tissues Center, in the Ukraine; Nu Tech Mediworld, in India; and Medra Inc., in the Dominican Republic. However, as there are no peer reviewed publications on Medline from these groups, nothing is known about how they prepare the cells, how the safety and side effects are evaluated, and how credible their claims are. There is a need to increase public awareness and to manage the public expectations for hESC based therapies. The ESC research programs being undertaken by corporations such as Geron Corporation, CA, USA, Advanced Cell Technology, CA, USA pave the way for better planned and transparent procedures employed embryonic stem cell therapy [64,65]. However, extensive research is still required to streamline hESC differentiation and develop cell transplantation methodologies. The recent reports of reprogrammed adult cells yielding the induced pluripotent cells may put an end to the ethical debates over the use of human oocytes to create stem cells [12,66]. Also the derivation of embryonic stem cell lines that are "HLA-homozygous," now provides avenues to overcome the problem of immune rejection and may pave way to utilize ESCs as therapeutics [67]. Trivedi et al., (2006) has reported a unique technique for tolerance induction using nuclear transfer (NT)-hESC-induced hematopoietic chimerism with synergistic use of adult bone marrow [68]. This nuclear transfer (NT)-hESC line was derived by transferring a donor cumulus cell into an enucleated oocyte, and subjected to electrical fusion followed by culture for 5 days. Although these reports are very promising a great deal of preclinical research still needs to be undertaken before the envisioned therapeutic potential of ESCs can be translated to the bedside. With more number of countries involving themselves in human ESC research it is expected that the progress will be faster and the technology may be brought to a clinical platform sooner than we may predict.

## Abbreviations

hESCs: Human embryonic stem cells; CRTs: Cell replacement therapies; ICM: Inner cell mass; IVF: In vitro fertilization; SCNT: Somatic cell nuclear transfer; PGDs: Preimplantation genetic diagnosis; BLSCs: Blastomere-like stem cells; ELSCs: Embryonic-like stem cells; FACS: Fluorescence Activated Cell Sorting; ART: Assisted reproductive technologies; hNT-ESCs: Human nuclear transfer ESC.

## Competing interests

The author(s) declare that they have no competing interests.

## Authors' contributions

KD has written the initial manuscript and compiled the information. KS has added the references and recent updates in the area. Both KD and KS have revised and read the manuscript before final approval. All authors have read and approved the final manuscript.
